# Emotional Picture and Word Processing: An fMRI Study on Effects of Stimulus Complexity

**DOI:** 10.1371/journal.pone.0055619

**Published:** 2013-02-11

**Authors:** Lorna H. Schlochtermeier, Lars Kuchinke, Corinna Pehrs, Karolina Urton, Hermann Kappelhoff, Arthur M. Jacobs

**Affiliations:** 1 Cluster of Excellence “Languages of Emotion”, Freie Universität Berlin, Berlin, Germany; 2 Department of Education and Psychology, Freie Universität Berlin, Berlin, Germany; 3 Department of Philosophy and Humanities, Freie Universität Berlin, Berlin, Germany; 4 Experimental Psychology, Department of Psychology, Ruhr-Universität Bochum, Germany; 5 Dahlem Institute for Neuroimaging of Emotion (D.I.N.E.), Freie Universität Berlin, Germany; University of Leuven, Belgium

## Abstract

Neuroscientific investigations regarding aspects of emotional experiences usually focus on one stimulus modality (e.g., pictorial or verbal). Similarities and differences in the processing between the different modalities have rarely been studied directly. The comparison of verbal and pictorial emotional stimuli often reveals a processing advantage of emotional pictures in terms of larger or more pronounced emotion effects evoked by pictorial stimuli. In this study, we examined whether this picture advantage refers to general processing differences or whether it might partly be attributed to differences in visual complexity between pictures and words. We first developed a new stimulus database comprising valence and arousal ratings for more than 200 concrete objects representable in different modalities including different levels of complexity: words, phrases, pictograms, and photographs. Using fMRI we then studied the neural correlates of the processing of these emotional stimuli in a valence judgment task, in which the stimulus material was controlled for differences in emotional arousal. No superiority for the pictorial stimuli was found in terms of emotional information processing with differences between modalities being revealed mainly in perceptual processing regions. While visual complexity might partly account for previously found differences in emotional stimulus processing, the main existing processing differences are probably due to enhanced processing in modality specific perceptual regions. We would suggest that both pictures and words elicit emotional responses with no general superiority for either stimulus modality, while emotional responses to pictures are modulated by perceptual stimulus features, such as picture complexity.

## Introduction

Most studies investigating emotional information processing use either verbal or pictorial stimuli to induce emotion, reliably revealing the involvement of limbic and paralimbic regions, such as the amygdala, the hippocampus, the medial prefrontal cortex, the anterior cingulate, the insula, or the visual cortex (e.g. [1]–[3]). It has been claimed that pictorial stimuli are able to induce higher emotional reactions, but it still remains an open question whether it is indeed the modality that is responsible for stronger emotional responses or whether the previously found superiority of pictures may rather be attributed to perceptual stimulus features such as differences in perceptual complexity, i.e. the amount of visual details of a stimulus.

Differences in the processing of verbal and pictorial information have been discussed extensively in the past without being explained conclusively. While some authors claim that pictorial and verbal information is processed in much the same way [4], dual coding theories (e.g. [5], [6]) postulate that information in pictures and words is processed differently and along distinct channels, thus creating separate semantic representations. Arguing for shared information representation and similar processing of pictures and words, Caramazza [4] claimed that semantic information is represented in a functional unitary system that is directly accessed by both visual objects and words. In contrast, Glaser postulated a distinction between a semantic system to which pictures have a privileged access and a lexicon, which includes only linguistic knowledge (e.g. [6], [7]). More recent influential theories of semantic processing propose that meaning is represented as embodied simulations of previous experiences (e.g. [8]), suggesting a unitary experience-based representation system.

While the theories, which try to explain differences between picture and word processing, are still underdetermined, the neuropsychological and neuroimaging data is equally inconclusive. Either the involvement of different networks, supporting a dual coding perspective (e.g. [9]–[11]), or high concordance in early processing of words and pictures [12], [13] and a shared semantic network, involved in the processing of words and pictures are reported, with few modality-specific areas [14]. However, a common finding is an observed processing superiority of pictures as compared to words (e.g. [10], [11]), suggesting that pictures have a faster and more direct access to meaning, while words are discussed to require additional translational activity at the representational level before accessing the semantic system.

Rather than trying to solve this ongoing debate, the present study focuses on the processing of emotional information in pictures and words. Here the literature is sparse with only few studies having compared both stimulus categories. In a behavioral study, DeHouwer and Hermans [15] found that emotional pictures, but not words, produced interference effects in a word–picture affective Stroop task. Also, naming times were faster for negative pictures, but not for negative words. In line with theories of dual coding [5], [6], these authors concluded, that pictures also have a privileged access to emotional information, which they suggest is represented in the same system as semantic information. Recent event-related potential (ERP) studies, on the other hand, revealed that emotional words and faces [16] as well as emotional words and pictures [17] may be decoded by the same cortical system, but at different processing speeds. The existent ERP evidence hence seems to support a notion of emotional pictorial stimuli that are processed with superiority, regarding processing speed, as compared to symbolic word stimuli, while probably using similar brain areas [16], [17]. In an fMRI study, Kensinger and Schacter [18] found processing differences with an overall superiority, i.e. stronger and more widespread activations, for pictures as compared to words, as well as a lateralization of responses between the modalities in the amygdala. The authors presented positive, negative, and neutral words as well as pictures taken from the International Affective Picture System (IAPS, [19]) in a semantic categorization task. Both, emotional words and pictures showed enhanced activity of several regions of the prefrontal and anterior temporal cortex, and in occipital visual processing regions, while the amygdala showed a lateralized emotion effect with left-lateral activations for words and more pronounced bilateral activations for pictures. This study thus suggests an overall superiority regarding the strength of activations for the pictures.

The observed pictorial superiority in processing speed and strength of activations might be accounted for by the involvement of different neural systems as suggested above [15]. Nonetheless, the alternative perspective, that semantic content and its emotional valence is represented in a unitary system, which is accessed by both pictorial and verbal information, might also apply. While the previously observed differences in processing speed could be attributed to translational activities necessary for the words, as suggested above [16]–[18], we propose an alternative explanation: Some of the previously observed processing advantages, regarding the strength of activations of emotional pictures, may be attributed to differences in perceptual complexity or the amount of visual information of the stimuli rather than to distinct modality-specific processes. As pictorial stimuli are characterized by more complex visual features than words, and since pictures and words are expected to be processed in parallel in early perceptual processing stages, the pictorial stimuli might be able to activate more semantically related details and memories. Given that higher semantic complexity in itself has been suggested to be a constitutive part of emotional representations (the ‘semantic cohesiveness’ hypothesis, [20], [21]), it can be assumed that such differences in terms of amount of detail, color, and discriminability between lexical and pictorial stimuli accounts for many of the observed processing differences. The potential effect of visual complexity in emotion processing has indeed been emphasized in the past (e.g. [22], [23]), but to our knowledge the impact of visual complexity of emotional pictures or words has not yet been studied directly. However, examining the neural correlates of visual complexity in the processing of abstract non-emotional, visual stimuli, Jacobsen et al. [24] observed parametric increases with increasing visual complexity in right hemisphere anterior and dorsolateral prefrontal regions and the bilateral fusiform gyrus. Remarkably, these regions also revealed higher activation for emotional pictures in the Kensinger and Schacter study [18].

The goal of the present study was to examine differences and similarities in the processing of emotional verbal and pictorial information and the role of stimulus complexity for explaining previous results. Therefore, the processing of pictorial and verbal emotional stimuli was compared in a valence judgment paradigm, while physical differences were maximally controlled. For this, concrete neutral and positive objects were presented in different modes: (a) as pictorial stimuli, we used visually reduced but still complex photos, as well as visually highly reduced black and white pictograms; and (b) as verbal stimuli, we used complex adjective-noun-phrases, as well as single words, so that both stimulus modalities (pictorial and verbal) were presented in two levels of complexity. Based on the notion of a unitary representational system and proposing the existence of a common emotional system, we expected that pictorial and verbal emotional stimuli would reveal comparable emotional effects when pictorial stimuli are reduced in visual complexity. Common emotional effects for both modalities should be revealed in emotion processing networks, i.e., the amygdala, the hippocampus, the anterior cingulate cortex, occipital regions, and in the medial prefrontal cortex, with no significant differences between stimulus modalities in subcortical and frontal emotional regions. Moreover, emotional effects should be modulated by the complexity manipulation, leading to stronger effects for the more complex stimuli for both the verbal and pictorial materials.

## Methods

### Participants

Twenty-one right-handed, healthy young German native speakers (male: 8, female: 13; age: mean = 24.29, SD = 3.481 (ranging from 18 to 32) participated in the study. Participants had normal or corrected to normal visual acuity and no known neurological condition. After being informed about potential risks and screened by the study psychologist, participants gave written informed consent before participating. The experimental standards were approved by the German Psychological Association (DGPs, the Society) ethics committee. Data were handled anonymously. Some of the participants received course credits; others were remunerated at the rate of 8 Euro per hour.

### Material

A total of 160 positive and neutral concrete objects were presented as stimuli, half of which as words and as pictograms, the other half as phrases and as photos. Thus, the whole experiment comprised the presentation of 40 positive and 40 neutral words, 40 positive and 40 neutral phrases, 40 positive and 40 neutral pictograms, and 40 positive and 40 neutral photos, while words and photos, and pictograms and phrases, respectively, referred to the same objects. Per stimulus category 4 additional negatively valenced stimuli were used as filler items.

Words were taken from the Berlin Affective Word List Reloaded (BAWL-R; [5]), pictograms from the “International Picture Naming Project at CRL-UCSD” (IPNP; [26]). Photos were selected to only show the object on a white background. Three-word-phrases were constructed by extending the concrete word by an article and an adjective. In order to produce comparability, only those photos and pictograms whose German expression matches the BAWL-R label were included leading to a database of 235 photos, pictograms, phrases, and words each. In a pre-study, all photos and pictograms were rated for valence, arousal and visual complexity, all words and phrases for valence, arousal and imageability by at least 20 participants. The final stimulus material was carefully selected so that in each stimulus condition the 40 positive and neutral items only differed in their mean valence rating (all p’s<0.001). In addition, photos and pictograms were matched in arousal and visual complexity between positive and neutral items (all p’s>0.34). Positive and neutral phrases were matched for arousal, imageability and length (all p’s>0.28), while words were matched for arousal, imageability, word frequency, number of letters, number of phonemes, and number of orthographic neighbors (all p’s>0.26).

### fMRI Experiment

**Figure 1:**
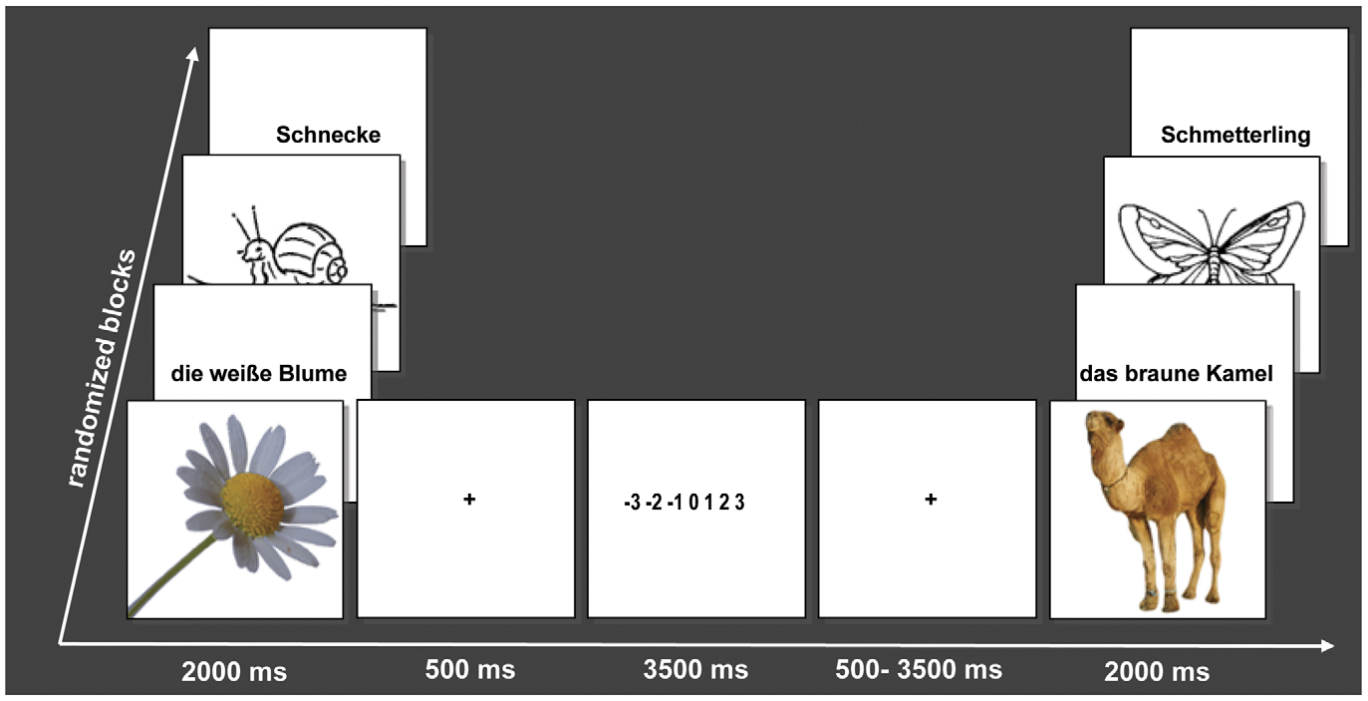
Experimental design with examples of stimuli (photos, pictograms, phrases and words; Schnecke = Snail; die weiße Blume = the white flower; Schmetterling = Butterfly; das braune Kamel = the brown camel).

Stimuli were presented in the scanning session using goggles with Presentation 12.1 software (Neurobehavioral Systems Inc.) in randomized order, pictograms and photos in 300×300 pixel resolution, placed in the middle of the screen, verbal stimuli using font type “Arial”, size 40, both black on a blank white screen. Each trial (see Figure 1) began with the stimulus that lasted for 2000 ms on its own, followed by a fixation cross (+) presented for 500 ms and then followed by a valence judgment task, consisting of a 7 stepped rating scale lasting for 3500 ms, ranging from −3 (very negative) via 0 (neutral) to +3 (very positive). This was then followed by a fixation cross and a randomly jittered inter-trial interval (average 2500 ms), used to sample the hemodynamic response at different time points (trial duration = 8500 ms – 11500 ms). Participants viewed 84 stimuli in each stimulus type, with 40 positive, 40 neutral and 4 negative filler items per stimulus type, presented in two runs with four blocks each in randomized order, so that the whole experiment lasted 47 minutes.

### Data Acquisition

Participants were informed about the experimental task and the scanning procedure before the scanning session. Before the experimental session a block of ten trials, which were not part of the stimulus set, was presented, to let the participants become acquainted with the task. The participants were instructed to use their right index finger to press the button and the palm of the hand to scroll a tracking ball. Form fitting cushions were used to prevent head movements. Earplugs and headphones were provided to attenuate scanner noise. After the scanning the stimuli were presented again on a computer screen and were rated for arousal. Imaging was performed with a 3T Siemens (Erlangen, Germany) Tim Trio MRI scanner equipped with a 12-channel head coil at the D.I.N.E. (Dahlem Institute for Neuroimaging of Emotion). In each of the two runs 740 whole-brain functional T2*weighted echoplanar (EPI) (TR: 2000 ms, TE: 30 ms, 90°Flip Angle 37 slices, matrix: 64×64, field of view (FOV): 192 mm; 3×3×3 mm voxel size, no gap) were acquired. Additionally, a T1-weighted matched-bandwidth high-resolution (voxel-size 1.0×1.0×1.0 mm) anatomical scan with same slice prescription as EPI was acquired.

### Data Analysis

The MRI data were processed using SPM 8 (Available: http://www.fil.ion.ucl.ac.uk/spm/, Accessed: 2013 January 11.). The images were slice-time corrected, realigned to the mean volume, spatially normalized to the standard EPI template provided by the Montreal Neurological Institute (MNI template). Then images were spatially smoothed with an 8 mm full-width at half-maximum (FWHM) Gaussian kernel. After preprocessing, the data were analyzed in an event-related design time-locked to stimulus onset in the context of the General Linear Model (GLM) as implemented in SPM 8 on two levels. In a first GLM analysis the experimental conditions of positive and neutral photos, pictograms, phrases and words were included as regressors in the design. On the second-level a 4 (stimulus type) × 2 (valence) repeated-measurement-ANOVA was calculated to assess effects of valence and stimulus type (modality and complexity). To further specify common and differential valence effects between the modalities, conjunction and interaction analyses were performed. For the conjunction, a method implemented in SPM 8 using a global null hypothesis as described by Friston et al. [27], which is based on the calculation of a minimum T-statistics, as it was originally introduced by Worsley and Friston [28]. Subsequently, to retrieve additional information of valence and complexity that go beyond the categorical distinction, a second parametric GLM analysis was calculated in which a single experimental condition effect was incorporated on the first-level, that included individual valence ratings and item-specific stimulus complexity, as well as the interaction between valence and complexity, as mean-centered parametric modulators in the design. Stimulus complexity was defined as jpeg comprimation size, of each respective stimulus, which has previously been used as an objective measure of image complexity [29]. The acronym jpeg refers to an international digital still image compression standard and stands for Joint Photographic Experts Group [30]. According to this encoding standard, the jpeg file size increases with increasing image components, if the image resolution is constant. In this way, the height of the expected hemodynamic response was parametrically adjusted for all events as a function of each subject’s valence ratings and each stimulus’ visual complexity as well as the interaction of both. In SPM the modulators are orthogonalized according to the sequence in which they are added in the design [31]. On second level one-sample t-tests were conducted to investigate brain activations that had a linear relationship as a function of individual valence ratings, visual stimulus complexity as well as of the interaction of valence ratings and complexity. For valence an overall effect was calculated. Otherwise all analyses were conducted separately for the pictorial, comprising the photos and pictograms, and the verbal stimuli, comprising the words and phrases, to examine the simple effects with their specific response patterns.

To protect against false-positive activations in both analyses, only clusters with more than 10 voxels were considered. All reported activations for the main effects survived a threshold corresponding to p<0.001, uncorrected.

Behavioral data were analyzed with SPSS 13 (SPSS 13.0 for Windows). Correlation analyses and repeated measures analyses of variances with reaction times, valence ratings, arousal ratings and complexity as dependent variables and stimulus type, modality, valence categories as independent variables were conducted.

## Results

### Behavioral Results

There was no reaction time difference in the valence judgments between verbal and pictorial materials (p = 0.120), and no significant reaction time difference in the valence judgments between positive and neutral stimuli (p = 0.499). Although our stimuli were matched for arousal, valence, and imageability in each stimulus condition in a pilot study, the inner-scanner ratings of the stimuli revealed differences in mean valence ratings for positive stimuli between verbal and pictorial stimuli (mean verbal = 1.17, SD = 0.443; mean pictorial = 0.935, SD = 0.460; F(1,79) = 14.927, p = 0.000). Post-scanning ratings of arousal revealed no differences in arousal between stimulus types (p = 0.683). Stimulus types differed in mean complexity, the respective jpeg picture comprimation size showing a significant main effect (F(3.319) = 152.137; p = 0.000) between stimulus types, with photos (mean = 22.666, SD = 10.114) being the most complex, followed by pictograms (mean = 14.895, SD = 6,310), phrases (mean = 7.530, SD = 1.087), and words (mean = 3.992, SD = 1.050). Valence ratings were correlated with stimulus complexity only for phrases (Pearson’s correlation = 0.224, p = 0.045), while no other significant correlations were observed (all p values >0,287).

### Imaging Results

#### Overall and simple effects of positive valence

**Figure 2:**
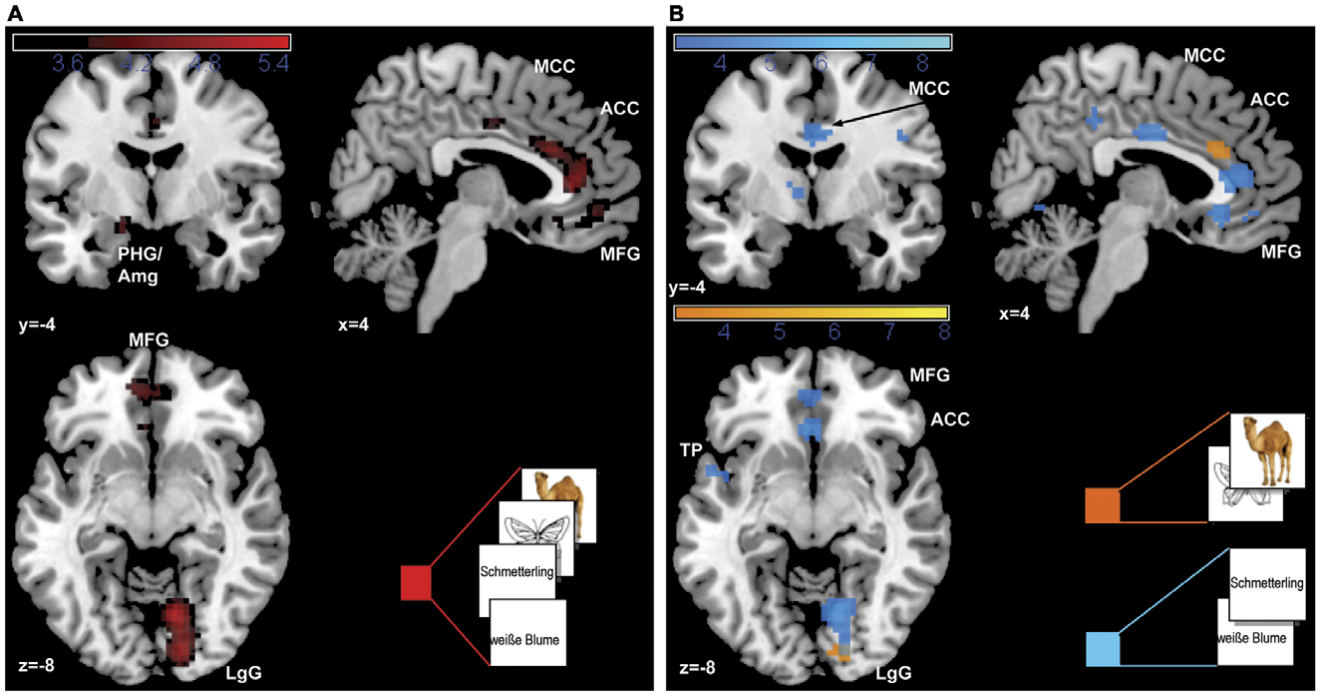
Main effects of positive valence. Activations for (A) all stimuli and (B) simple effects for pictorial and verbal material are presented at p<0.001. Abbreviations: Amg = amygdala; ACC = anterior cingulate cortex; LgG = lingual gyrus; MCC = mid cingulate cortex; MFG = medial frontal gyrus; PHG = parahippocampal gyrus, TP = temporal porle.

**Figure 3:**
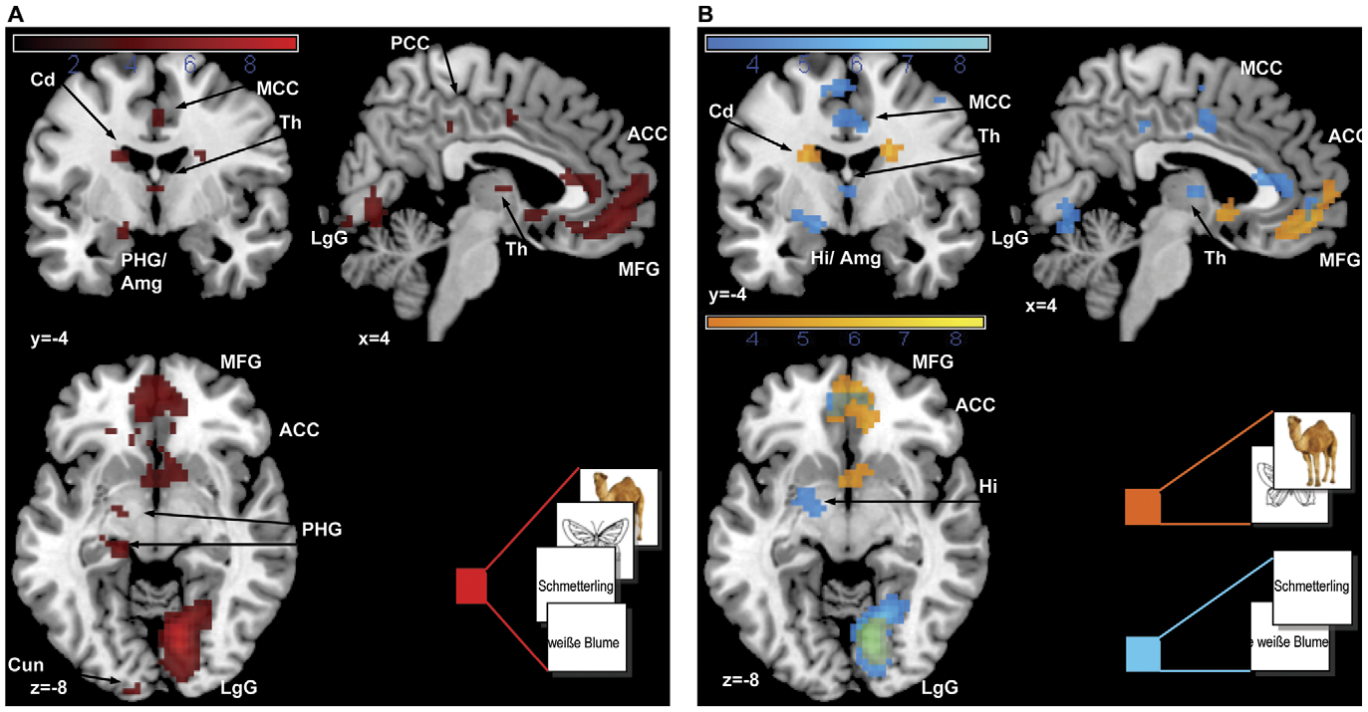
Parametric main effects of subjective valence ratings. Activations for (A) all stimuli and (B) simple effects for pictorial and verbal material are presented at p<0.001. Abbreviations: Amg = amygdala; ACC = anterior cingulate cortex; Cd = caudate nucleus; Cun = cuneus; Hi = hippocampus; LgG = lingual gyrus; MCC = mid cingulate cortex; MFG = medial frontal gyrus; PHG = parahippocampal gyrus; PCC = posterior cingulate cortex; Th = thalamus.

As depicted in Table 1 and Figure 2 the main overall effect of positive valence revealed activations in an emotion processing network including the right anterior cingulate cortex (BA 24), the left frontal pole (Brodmann Area, BA 10), as well as the left parahippocampal gyrus extending to the amygdala, and in visual processing regions, namely the right lingual gyrus and the left cuneus (BA 18). To differentiate between modalities, the simple valence effects were analyzed separately for pictorial and verbal materials revealing, for the pictorial materials (photos and pictograms), significant activations for positive valence in the right lingual gyrus (BA 17), as well as in the right anterior cingulate (BA 32) and left frontal pole (BA 10). For the verbal material (phrases and words) widespread activations were observed in frontal emotion processing regions including the right anterior cingulate cortex (BA 24), the left frontal pole (BA 10), the left insula (BA 13), and the left caudate tail, as well as in language processing regions: the left precentral gyrus (BA 4), the thalamus, and the left temporal pole (BA 38). In the parametric analysis, effects for individual valence ratings as a linear function of increasing valence were found for the overall effect in a similar but more widespread network including the lingual gyrus, the bilateral frontal cortex (BA 11, 10, 8), the left anterior cingulate cortex (BA 24, BA 25), the left caudate body and the left parahippocampal gyrus, extending to the amygdala. In the simple effects besides activations in the left medial frontal gyrus and the right anterior cingulate, additional activations for the pictorial material were found in the left caudate body and the subgenual part of the anterior cingulate as depicted in Figure 3. For the verbal material the network was more extensive including the right anterior cingulate cortex (BA 32), the left middle frontal gyrus (BA 8), the left caudate body, the right insula (BA 13), the right precentral gyrus (BA 4), and the thalamus with additional activations in the left transverse temporal gyrus, the left hippocampus extending to the amygdala, in the left cerebellar declive, the right inferior frontal gyrus (BA 47), and the left superior parietal lobe (BA 7) (see Table 1, Figure 3).

**Table 1:** Anatomical locations, hemisphere (left/right, L/R), brodmann area (BA), mni (Montreal Neurological Institute) coordinates, cluster size (Voxel) and T-scores of activation maxima at p<0.001 and cluster size >10 for main effects of valence.

Anatomical area	L/R	BA	MNI coordinates			Voxel	T score
			x	y	z		
Effects of positive valence							
*Overall*							
anterior cingulate cortex	R	24	6	35	7	180	4.97
medial frontal gyrus, frontal pole	L	10	−9	50	−5	141	4.78
midcingulate cortex	R	24	3	−4	40	14	3.71
parahippocampal gyrus, amygdala	L		−15	−7	−14	20	4.26
lingual gyrus	R	18	12	−67	−8	328	5.33
cuneus	L	18	−3	−100	7	28	4.01
*Pictorial*							
anterior cingulate cortex	R	32	0	29	25	32	4.20
medial frontal gyrus, frontal pole	L	10	−6	50	−5	14	3.78
lingual gyrus	R	17	15	−88	−8	23	3.79
*Verbal*							
medial frontal gyrus, frontal pole	L	10	−3	44	−8	27	3.59
anterior cingulate cortex	R	24	6	35	7	125	4.60
midcingulate cortex	R	24	0	−7	31	52	4.24
	R	31	3	−37	40	14	3.62
precentral gyrus	R	4	48	−13	28	41	4.59
frontal lobe, white matter	L		−30	−13	34	12	3.65
paracentral lobule	L	31	−6	−28	46	12	3.37
insula	L	13	−36	−13	19	15	3.66
caudate nucleus, caudate tail	L		−36	−28	−2	10	3.71
temporal pole	L	38	−51	5	−8	15	3.68
thalamus	L		−9	−4	4	10	3.42
lingual gyrus	R	18	12	−67	−5	242	5.10
**Positive effects of individual valence ratings (parametric)**							
*Overall*							
medial frontal gyrus	R	10	3	50	−8	147	6.08
middle frontal gyrus	L	11	−24	32	−11	11	4.43
superior frontal gyrus	L	8	−24	32	49	86	5.45
anterior cingulate cortex	L	10/32	−6	53	1	644	6.26
posterior cingulate cortex	L	30	−9	−55	13	27	4.28
precentral gyrus	L	4	−48	−10	55	133	4.69
postcentral gyrus	L	40	−57	−22	16	62	4.73
postcentral gyrus	L	4	−12	−40	61	20	4.58
caudate nucleus, caudate body	L		−21	−7	22	23	5.07
parahippocampal gyrus	L	28	−18	−25	−11	40	5.82
parahippocampal gyrus, amygdala	L	28	−15	−4	−14	30	4.60
thalamus	R		3	−7	7	11	4.57
middle occipital gyrus	L	18	−24	−91	−2	17	4.47
cuneus	L	19	−6	−94	25	162	7.01
lingual gyrus	R	18	9	−67	−11	629	9.31
*Pictorial*							
medial frontal gyrus, frontal pole	L	10	−3	56	−2	381	6.68
medial frontal gyrus	L	8	−24	29	46	13	4.16
	R	11	9	35	−11	45	5.97
anterior cingulate cortex	R	25	0	8	−5	39	5.22
	R	24	9	26	10	13	4.81
caudate nucleus, caudate body	L		−21	−4	22	23	5.26
lingual gyrus	R	18	12	−76	−8	274	8.28
*Verbal*							
middle frontal gyrus	L	8	−27	32	49	48	6.47
inferior frontal gyrus	R	47	27	8	−17	17	5.23
anterior cingulate cortex	L	32	−9	47	−5	198	5.87
midcingulate cortex	L	31	−12	−25	43	727	6.74
precentral gyrus	R	4	30	−34	55	11	4.63
	R	4	36	−16	58	41	4.41
thalamus	L		−3	−13	7	32	5.37
precentral gyrus	R	4	51	−16	43	15	5.26
superior parietal lobe	L	7	−24	−55	58	29	4.87
transverse temporal gyrus	L	41	−57	−22	13	119	6.04
parahippocampal gyrus/amygdala	L	28	−15	−7	−11	43	5.51
insula	R	13	42	5	10	13	5.01
caudate nucleus, caudate body	L		−21	−10	22	14	4.66
white matter	L		−12	−58	16	20	4.26
cuneus	R	7	21	−82	28	26	4.13
lingual gyrus	R	18	12	−70	−8	723	8.68
cerebellum, declive	L		−27	−67	−17	25	5.02

#### Conjunction and interaction of simple valence effects

**Figure 4:**
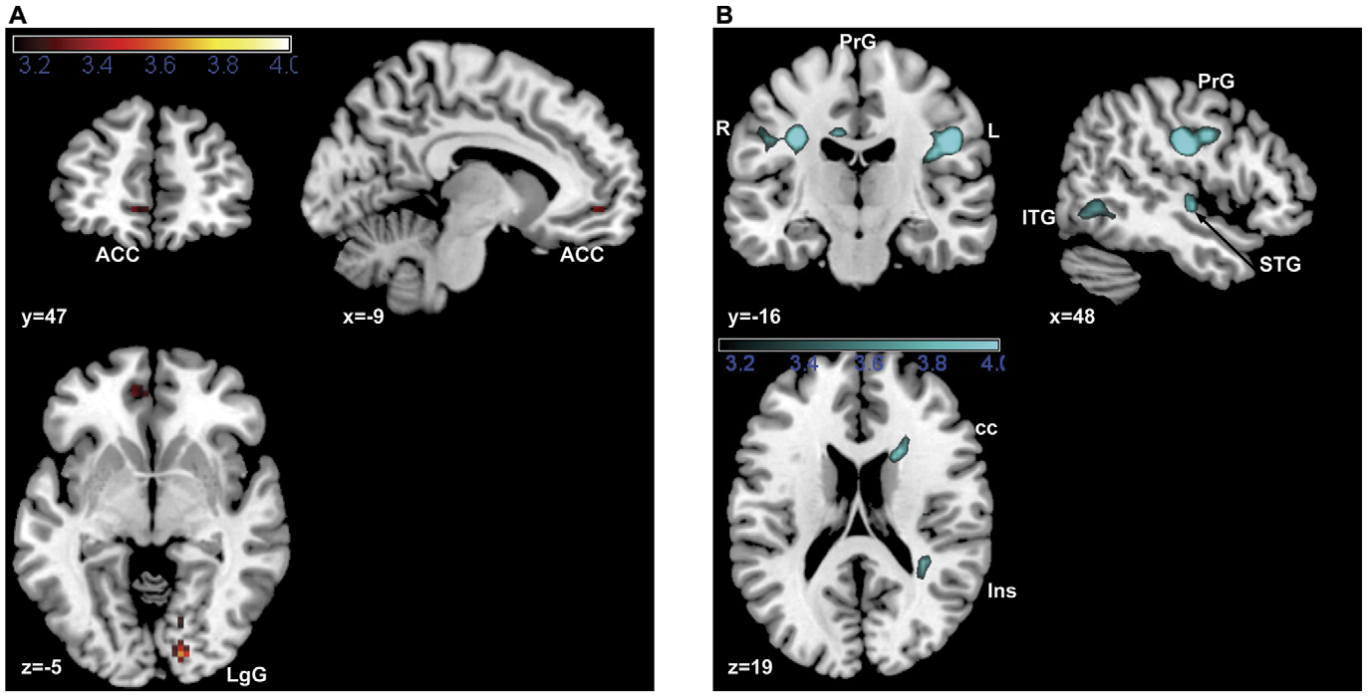
Conjunction of simple valence effects (A) is depicted and interaction effect of valence and modality is depicted only for (pos verbal>neut verbal)>(pos picorial>neut pictorial) (B), as there were no significant activations for (pos pictorial>neut pictorial)>(verb pos>neut verbal). Both are depicted at p<0.001. Abbreviations: ACC = anterior cingulate cortex; cc = corpus callosum; ITG = inferior frontal gyrus; Ins = insula; LgG = lingual gyrus; PrG = precentral gyrus (R = right; L = left); STG = superior temporal gyrus.

To identify common valence effects for the modalities a conjunction of the simple pictorial and verbal valence effects was analyzed, which revealed significant common activations in the right lingual gyrus and the anterior cingulate cortex (Table 2, Figure 4A). To compare valence effects between modalities, the interaction of valence and modality (verbal, pictorial) was examined, revealing increased activations for verbal material (Table 2, Figure 4B) in the right insula (BA 13) and the left midcingulate cortex (BA 31), as well as in a network of language processing regions including the right superior temporal gyrus, the right inferior temporal gyrus and the bilateral precentral gyrus (BA 3). No increased activations were found for the pictorial as compared to the verbal valence effects.

**Table 2:** Anatomical locations, hemisphere (left/right, L/R), brodmann Aarea (BA), mni (Montreal Neurological Institute) coordinates, cluster size (Voxel) and T-scores of activation maxima at p<0.001 and cluster size >10 for conjunction effect and interaction of valence and modality.

Anatomical area	L/R	BA	MNI coordinates			Voxel	T score
			x	y	z		
**Conjunction of verbal and pictorial valence effects**							
lingual gyrus	R	17	15	−88	−5	12	3.64
anterior cingulate	R	24	0	23	25	1	3.18
	L	32	−9	47	−5	15	3.32
**Interaction of valence and modality**							
*(pos verbal>neut verbal)>(pos pictorial>neut pictorial)*			–	–	–		
precentral gyrus	L		−30	−13	31	121	5.30
midcingulate cortex	L		−3	−4	31	60	4.26
precentral gyrus	R	3	48	−16	28	176	5.18
superior temporal gyrus	R	22	51	−10	−5	27	4.31
inferior temporal gyrus	R	19	48	−61	−5	38	3.67
Insula	R	13	33	−40	19	16	3.73
corpus callosum			18	14	22	21	4.16
			–	–	–		
(pos pictorial>neut pictorial)>(pos verbal>neut verbal)			–	–	–		

#### Simple interaction effects of valence and complexity

The role of visual complexity for emotion processing was examined separately in verbal and pictorial stimulus material. In a first categorical analysis, stronger valence effects for the complex photos as compared to the simple pictograms were revealed in the right anterior cingulate cortex (BA 32), the left medial frontal gyrus (BA 11) as well as in the right middle occipital gyrus (BA 18) and the cuneus. The beta values, which are depicted exemplarily for the anterior cingulate cortex (Figure 5A, right), show an increase in activation from neutral to positive for the photos and a decrease for the pictograms. For verbal material no significant differences in valence effects were observed between words and phrases. In the subsequent parametric analysis, for the interaction of individual valence ratings and specific stimulus complexity, significant effects were found for the pictorial material only in the cerebellum. For the verbal material, again, no significant activation differences were observed. A visual inspection of the interaction parameters for these contrasts revealed that, for the pictorial stimuli, pictograms and photos overlap regarding high interaction parameter values (Table 3, Figure 5B, right). This indicates that the interaction effect is triggered by complex photos, but also by complex pictograms.

**Figure 5:**
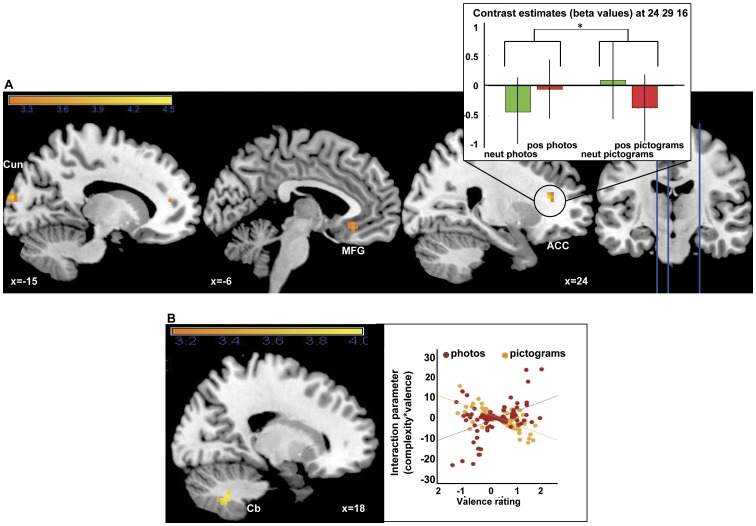
5. Simple categorical interaction effects of complexity and valence for pictorial stimuli (A) (pos photos>neut photos)>(pos pictograms>neut pictograms). Bar plot at right (A, top right) represents contrast estimates at peak voxels of the frontal poles. Simple parametric interaction effect of complexity and individual valence ratings (complexity × rating) for pictorial material (B). Plots at the right represent the interaction parameters in relation to valence ratings for (C) pictograms and photos. All activations are presented at p<0.001.Abbreviations: ACC = anterior cingulate cortex; Cb = cerebellum; Cun = cuneus; MFG = medial frontal gyrus.

**Table 3:** Anatomical locations, hemisphere (left/right, L/R), brodmann area (BA), mni (Montreal Neurological Institute) coordinates cluster size (Voxel) and T-scores of activation maxima at p<0.001 and cluster size >10 for parametric interaction effects.

Anatomical area	L/R	BA	MNI coordinates			Voxel	T score
			x	y	z		
Interaction of categorical valence and complexity							
*(pos photos>neut photos)>(pos pictograms>neut pictograms)*							
medial frontal gyrus	L	11	−6	29	−11	13	3.63
anterior cingulate cortex	R	32	24	29	16	11	4.18
cuneus	L	18	−15	−97	16	31	3.94
middle occipital gyrus	R	18	9	−94	10	67	4.39
(pos pictograms>neut pictograms)<(pos photos>neut photos			–	–	–		
*(pos phrases>neut phrases)>(pos words>neut words)*			–	–	–		
*(pos words>neut words)>(pos phrases>neut phrases)*			–	–	–		
Parametric interaction of valence ratings and complexity							
*Pictorial*							
cerebellum	R		18	−55	−41	54	4.75
*Verbal*			–	–	–		

## Discussion

The present study investigated the processing of positive emotional information and the role of visual complexity in verbal and pictorial material using a valence judgment task. Based on the notion of a unitary semantic representational system we proposed the existence of a common emotion system, and firstly hypothesized that an emotion network is involved in the processing of positive as compared to neutral stimuli with no relevant differences between the modalities. Secondly we expected stronger emotional effects for more complex stimuli, assuming that more detailed visual information would evoke enhanced emotional processing. In parts our observations supported these hypotheses. Both stimulus modalities activated an emotion network and, as expected, no superiority for pictorial stimuli in emotional processing was observed. An increase of emotional responses related to complexity was only found for pictorial, but not for verbal stimuli.

The first major question of this study concerned effects of valence in our perceptually controlled stimulus set and the differences in valence effects between the modalities. As expected, an overall effect of positive valence for all stimulus conditions revealed activations in a network of limbic and paralimbic regions associated with emotion processing including the parahippocampal gyrus extending to the amygdala, the frontal pole and the anterior cingulate. The amygdala has been associated with multiple roles in emotional processing and seems consistently involved in emotional enhancement of perception [32], [33], while the frontal pole has been proposed to play a specific role in the processing of positive valence [34]–[36], and the anterior cingulate cortex has been shown to be involved in valence judgment tasks [37], or more generally in affective decision making (e.g. [38], [39]). Apart from limbic and paralimbic areas, visual occipital regions were activated. Such co-activations of visual regions with the amygdala are consistent with previous findings and can be attributed to the involvement of the amygdala in emotional enhancement of visual processing [32], [33], [18]. The results of the parametric effects of individual valence ratings revealed a similar pattern, while activations here were stronger and more widespread.

Interestingly, the differential valence effects for verbal and pictorial stimuli are at odds with the previously reported superiority of pictures over words [18]. In our data no reaction time differences between pictorial and verbal stimuli were observed and in contrast to previous studies the valence effects for pictorial stimuli actually appeared less strong and less widespread than for the verbal material. While common activation patterns of both modalities were observed in the anterior cingulate cortex and the frontal pole as well as in occipital regions, our data also revealed more widespread activations for the verbal material, with additional activations in the insula, the subgenual part of the anterior cingulate, the caudate tail, and the midcingulate cortex. These regions have previously been associated with emotion processing and response selection [40]–[42]. Further valence dependent activations for verbal stimuli were observed in language processing regions, in the precentral gyrus and the left temporal pole, which is in line with previous findings of reading emotional words [3]. This might be due to effects of emotional enhancement of language perception and evaluation. The parametric analysis of the effects of individual valence ratings supports these findings by showing similar but slightly more widespread patterns of activations for both pictorial and verbal material. For the pictorial stimuli significant activations were now also found in regions that had in the categorical analysis only been activated in the verbal condition, namely in the caudate nucleus and the subgenual part of the anterior cingulate. The verbal stimuli revealed additional significant activations in the left amygdala as well as in the superior parietal lobe, the transverse temporal cortex, the cerebellum, and the inferior frontal gyrus (BA 47), which are associated with language and semantic processing [43], [44]. Finding a significant activation in the left amygdala only in the verbal condition might suggest a lateralization of emotional responses related to the modality and to functional differences between emotional words and pictures, as it has previously been reported by Kensinger and Schacter [18].

Confirming what is already visible in the simple differential emotional effects, overlapping emotional effects in occipital regions and in the anterior cingulate cortex were revealed in the conjunction analysis. In the interaction analysis, then, neither stronger valence effects for the pictorial as compared to the verbal stimuli, nor any activation differences in the amygdala or other subcortical emotion processing regions were found. Thus, our pictorial stimuli did not show the previously reported superiority in emotional processing [18]. In contrast, increased activations were observed for the verbal stimuli, mainly in language processing regions, such as the superior temporal gyrus, the inferior temporal gyrus and the precentral gyrus. These may be attributed to feedback-projections from subcortical regions to modality-specific perceptual regions. Some of these activations were found in the right hemisphere though. However, while there is ample evidence in the literature for a dominance of the left hemisphere in language processing, the right hemisphere has also been shown to contribute to language processing, for example when concrete words are processed [45], which is the case in this experiment.

Considering, that the main novelty of this study regarded the reduction of complexity in the pictorial material in comparison to previous studies, not finding any superiority of responses for the pictures, but rather increased valence effects for the verbal stimuli, already indicates that visual complexity contributed to the previously observed processing superiority of emotional pictures. To better understand the role of complexity in our data, we looked at the interaction of valence and complexity. This revealed as hypothesized, that stronger valence effects were indeed associated with higher visual stimulus complexity, but differently for the two modalities, pointing to an important role of stimulus complexity in visual emotional information processing. We find, in accordance with our hypothesis, stronger effects for complex photos as compared to simple pictograms, whereas –unexpectedly - no effects of complexity in the verbal material, when comparing valence effects between complexity categories, words and phrases. Photos as compared to pictograms activated a network of emotion processing and evaluation in the medial frontal gyrus and the anterior cingulate cortex, as well as primary visual processing regions, the middle occipital gyrus and the cuneus. The results of the parametric analysis on the other hand only showed a significant activation in the cerebellum. Considering the overlapp of interaction parameters for photos and pictograms, the fact that photos, besides being more complex, are also more colorful and realistic than pictograms, may partly explain their stronger emotion effects. Indeed, the role of color for emotional responses has been discussed before. Some authors found differences in early processes of the electro-cortical response [46], [47], while several others presented evidence against a role of color for the emotional response, or for only small effects of color [48], [49]. Another explanation might be, that since pictures are 2-dimensional images of the real world and as photos are more realistic than pictograms, their realistic quality might contribute to the elicited emotional response. Color might then also be considered as one potential property contributing to the higher realistic quality of photos. For the verbal material, in the parametric analysis, as in the categorical analysis, no differences related to complexity were observed. Thus, as expected, the higher amount of visual information in the photos triggered increased perceptual and semantic processing associated with stronger responses in a semantic and emotion processing network. In the verbal material, on the other hand, complexity did not show an influence on the emotion effect.

## Conclusions

At odds with previous findings, we find no stronger or more widespread emotion effects for pictorial than for verbal stimuli. Differences in emotional processing between the modalities were mainly found in language processing regions and might mainly be due to feedback projections to perceptual regions. A role of visual complexity and amount of visual information in the stimuli for the intensity of the emotional reaction was present for the pictorial stimuli, but more so in the categorical analysis, speaking for differences between photos and pictograms, such as color or realistic quality, that, besides complexity, may also play a role for the intensity of the emotional response.

It should be noted, that the present study bears some limitations in that it was restricted to positive stimuli, and the results would thus have to be replicated for stimuli of negative valence and an independent manipulation of stimulus arousal. Despite these limitations, our study gives clarification to the debate on differences of emotional picture and word processing. It provides evidence, that there is no general superiority of emotional responses for either verbal or pictorial stimuli, while showing that visual complexity might partly account for the previously found processing differences and stronger effects of emotional pictures. Our results thus do not speak for a central difference in emotional information processing between modalities, and an advantage for pictures, as proposed by deHouwer and Hermans [6], [15]. However, they also do not suggest, that semantic content is the sole factor determining the emotional response, as assumed by theories proposing a functional unitary representation and emotion system [5]. Pictorial and verbal stimuli seem to share a common network of emotion processing, while some processing differences seem to exist. These might mainly be attributed to modality-specific emotional enhancement of perceptual processes, as they were mainly found in brain regions associated with language processing. The fact, that the left amygdala was only significantly activated for the verbal stimuli might indicate some lateralization of emotion effects related to the modality. However this lateralization effect was not significant in the interaction analysis and could thus also be attributed to the overall stronger effects in the verbal condition.

Therefore we would support a view of a common emotional system for pictures and words, together with some processing differences, which we suggest are mainly related to stimulus-specific, emotionally enhanced perceptual processing. A theory of an experience-based semantic representation system might apply, in which concrete symbolic information is re-experienced from memory while pictorial information, being more realistic, is experienced more directly [8] and is consequently more dependent on perceptual stimulus features such as visual complexity. Still, both stimulus modalities would activate overlapping embodied representations of emotional meaning.

Studies using stimuli to evoke emotions should take into account the variability of responses to pictorial material due to perceptual properties. Pictorial stimuli seem to have the advantage of being more realistic; verbal stimuli on the other hand have an advantage in controllability, while, although highly symbolic, they are able to evoke comparably strong emotional responses. As the present study was only designed to examine effects of visual complexity, future research should further investigate the processes involved in the evaluation of emotional information related to other perceptual stimulus features, such as realistic quality. Apart from the modulation of emotional responses by visual complexity, it remains an open question how emotional meaning is represented in general and how it is accessed, given how realistic or symbolic a stimulus is.
